# *POLG* R964C and *GBA* L444P mutations in familial Parkinson's disease: Case report and literature review

**DOI:** 10.1002/brb3.1281

**Published:** 2019-04-02

**Authors:** Pei‐Chen Hsieh, Chun‐Chieh Wang, Chia‐Lung Tsai, Yuan‐Ming Yeh, Yun Shien Lee, Yih‐Ru Wu

**Affiliations:** ^1^ Department of Neurology Chang Gung Memorial Hospital at Linkou Medical Center Taoyuan Taiwan; ^2^ Genomic Medicine Core Laboratory Chang Gung Memorial Hospital Taoyuan Taiwan; ^3^ Department of Biotechnology Ming Chuan University Taoyuan Taiwan; ^4^ Department of Neurology, College of Medicine Chang Gung University Taoyuan Taiwan

**Keywords:** *GBA*, missense substitution, next‐generation sequencing, Parkinson's disease, *POLG*

## Abstract

Polymerase gamma (*POLG*) is an enzyme responsible for the replication and repair of mitochondrial DNA. Mutations in *POLG* may cause variable clinical manifestations, including parkinsonism, epilepsy, cerebellar ataxia, neuropathy, and progressive external ophthalmoplegia. However, mutations of this gene are rare in patients with typical Parkinson's disease (PD). We report a man (current age: 59 years) without any underlying disease presenting with right‐hand tremor at the age of 39 years, followed by slow movement, rigidity, and postural instability. He developed motor fluctuation and levodopa‐induced dyskinesia 8 years later. At the age of 58 years, cognitive decline and visual hallucination ensued; he was institutionalized thereafter. We used multiplex ligation‐dependent probe amplification, which demonstrated no large deletions or duplications of relevant PD genes. Next, targeted sequencing panel covering 51 genes causative for PD was applied for the proband; it revealed a heterozygous missense substitution R964C in *POLG* and a heterozygous missense substitution L444P in *GBA*. The patient's father, who had been diagnosed as having PD and type 2 diabetes mellitus at the age of 70 years, demonstrated identical mutations. This is the first report of familial PD combined with *POLG* R964C and *GBA* L444P mutations. Two pathogenic gene mutations potentially cause double hit in pathological neurodegeneration. This finding extends our understanding of the PD genotype–phenotype correlation.

## 1 INTRODUCTION

Parkinson's disease (PD) is the second most common neurodegenerative disease (Calabrese, 2007). With technological advancement, a growing list of genes have been confirmed to cause familial PD (Deng, Wang, & Jankovic, 2018; Lill, 2016; Puschmann, 2017). Next‐generation sequencing technology has been applied worldwide to identify the causative genes for various neurological disorders (Bahassi & Stambrook, 2014). The glucocerebrosidase gene (*GBA*) has been a candidate gene for PD for a decade (Deng et al., 2018). It is involved in lysosomal sphingolipid degradation. The heterozygous *GBA* L444P mutation is a high‐risk mutation for PD (O'Regan, deSouza, Balestrino, & Schapira, 2017). Moreover, polymerase gamma (*POLG*) is an enzyme responsible for the replication and repair of mitochondrial DNA (Chan & Copeland, 2009) and mutation in the *POLG* may cause various clinical manifestations, including parkinsonism (Miguel et al., 2014), epilepsy (Stricker et al., 2009; Stumpf, Saneto, & Copeland, 2013), cerebellar ataxia (Stricker et al., 2009; Stumpf et al., 2013), and progressive external ophthalmoplegia (Luoma et al., 2004; Miguel et al., 2014). R964C, a missense substitution *POLG* mutation, was considered to be related to manifestations of the central nervous system other than typical PD. Herein, we report the first case of a patient with young‐onset PD (YOPD) carrying both *POLG* R964C and *GBA* L444P mutations.

## 2 CASE PRESENTATION

A man (current age: 59 years), without any underlying disease, presented with a right‐hand tremor at the age of 39 years, followed by loss of facial expression, slow movement, rigidity, and postural instability. He also had rapid eye movement sleep behavior disorder (RBD), but no hyposmia or orthostatic dizziness. At the age of 45 years, his neurological examination revealed free ocular movement, no ptosis, and normal deep tendon reflex. He had right‐side predominant rigidity, bradykinesia, mild neck dystonia, and festinating gait. He generally responded well to levodopa. His Unified Parkinson Disease Rating Scale (UPDRS) III scores revealed more than 50% improvement under a levodopa equivalent dose of 790 mg. He then developed motor fluctuation and levodopa‐induced dyskinesia after 7 years of symptom onset. At the age of 58 years, he demonstrated progressive cognitive decline, visual hallucination, and was required to live in a nursing home. His Mini Mental State Examination score was 10 and his Clinical Dementia Rating was 2. We obtained patient's serum lactate and pyruvate level and all revealed normal. Nerve conduction study showed right deep peroneal motor axonal neuropathy and right ulnar nerve neuropathy cross elbow, which suggested an entrapment neuropathy. Eletroencephalogram revealed no epileptiform discharge. His father was diagnosed as having PD, along with type 2 diabetes mellitus, at the age of 70 years; his neurological examination revealed resting tremor, rigidity, and bradykinesia on the left side, all of which diminished after levodopa treatment. He had symptom of chronic insomnia, bilateral lower limbs pain, and anxiety but no RBD. Both patient and his family had no symptom of ataxia, proximal weakness, epilepsy, or ophthalmoparesis.

**Figure 1:**
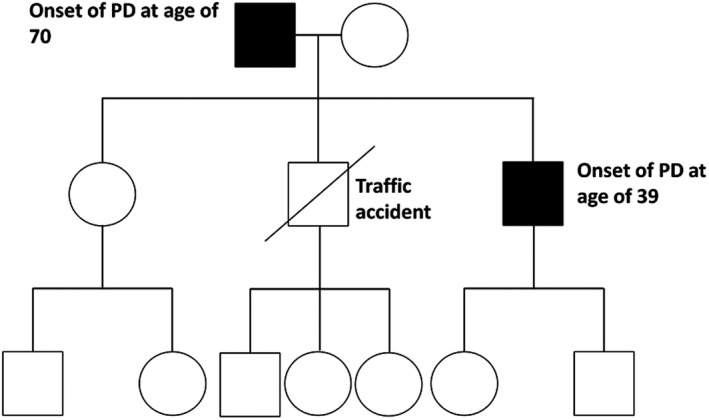
Family pedigree. Squares and circles represent males and females, respectively. Filled and slashed symbols indicate affected and symbols indicate deceased individuals

We could not obtain his mother's DNA sample because the patient had not been in contact with his mother and sister for many years. His younger brother died in a traffic accident without any history of parkinsonian symptoms. The rest of his family members did not have any extrapyramidal symptom, epilepsy, myopathy, or ataxia. Figure 1 presents the family pedigree of the patient's family.

Genomic DNA was extracted from peripheral venous blood lymphocytes of the patient and his father. Next, multiplex ligation‐dependent probe amplification was used to detect large deletions or duplication in the DNA (Jeuken, Cornelissen, Boots‐Sprenger, Gijsen, & Wesseling, 2006). We then used target exome sequencing with a TruSeq Custom Amplicon Low Input panel (Illumina) to determine the 51 PD‐causative genes mutation sites in patients (Deng et al., 2018; Lill, 2016; Puschmann, 2017). Target regions of patients' blood genomic DNA were amplified with specific primers, ligated of adaptors to the amplified PCR products, and finally generated the libraries. Paired‐end 150‐bp NGS were performed on an Illumina MiSeq system at the Genomic Medicine Core Laboratory, Chang Gung Memorial Hospital. The validation of NGS results was performed with automatic sequencer ABI 3730 (Thermo Fisher, USA).

Nonsynonymous single‐nucleotide polymorphisms, insertions–deletions, stop–gain, and frameshift variants were picked up. Next, Sorting Intolerant from Tolerant, Mutation Taster (http://www.mutationtaster.org/), and Polymorphism Phenotyping (version 2) were performed to detect amino acid substitutions affecting protein function. In addition, to determine potential candidate genes, we assessed the frequency of the variants in the general population (Exome Aggregation Consortium, dbSNP, 1000 Genomes Project). We considered variants with a minor allele frequency of ≤0.1% (rare variants). The mutation was classified as a pathogenic mutation if previous literature reported it as causative.

**Figure 2:**
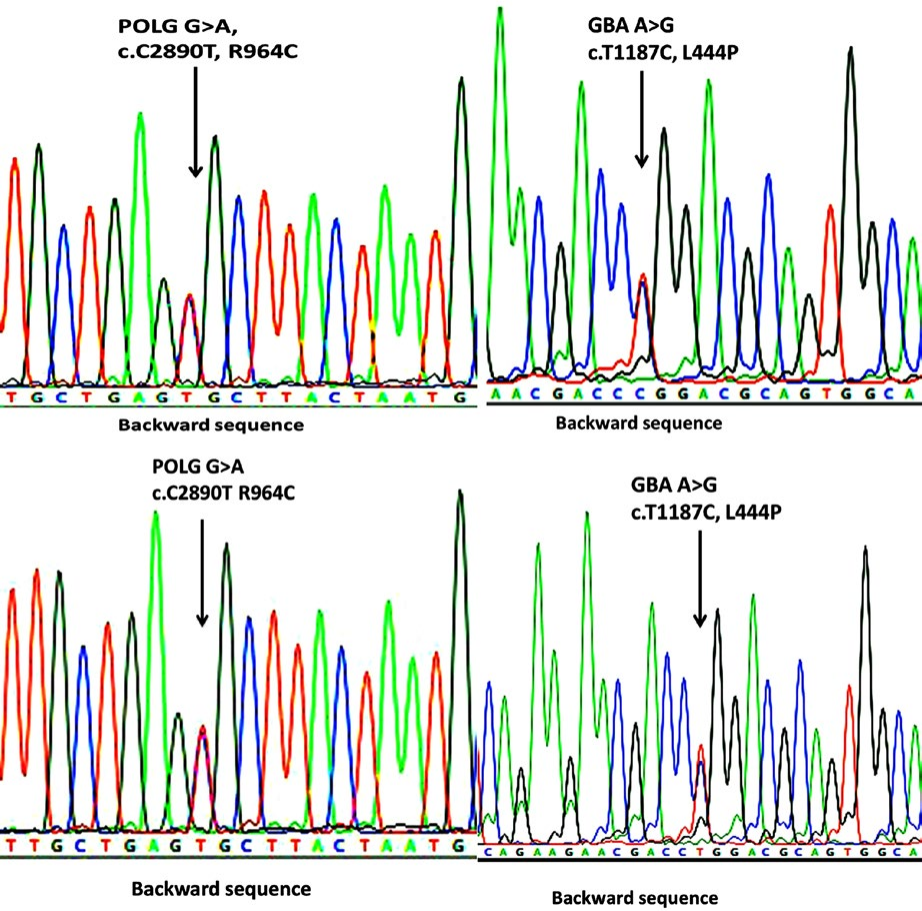
Sequence chromatograms showing the single nucleotide change in *GBA* and *POLG1*. The patient's (A and B) and his father's (C) and (D) chromatograms

**Figure 3:**
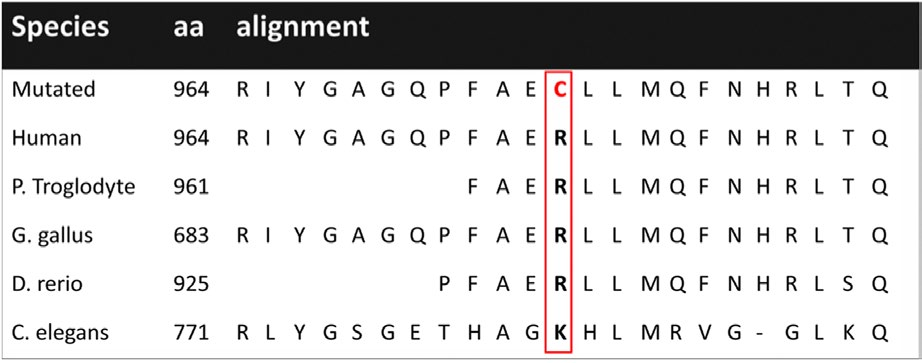
Mutant residues in *POLG1* and amino acid alignment showing evolutionary conservation of altered residues

We confirmed the presence of heterozygous missense substitutions in *POLG* [c.2890G > A (p.R964C)] as well as *GBA* [c.1187A > G (p.L444P)] in the patient and his father (Figure 2). *POLG* R964C signifies alteration in a highly conserved site (Figure 3). According to the American College of Medical Genetics guidelines, *POLG* R964C meets the pathogenic criteria as one strong pathogenic evidence, (PS3, well‐established in vitro functional studies supportive of a damaging effect on the R964C mutation), and two moderate pathogenic evidence (PM1, located in a mutational hotspot and functional domain without benign variation, and PM2, absent from controls (or at extremely low frequency if recessive) in Exome Sequencing Project, 1000 Genomes or ExAC. Combing the two criteria, this mutation is categorized as a likely pathogenic variant for PD according to the scoring rule (Richards et al., 2015). Moreover, *GBA* L444P is categorized as a pathogenic gene in the ClinVar database.

## 3 DISCUSSION

Thus, our patient and his father demonstrated a typical presentation of idiopathic PD, with two mutation sites in *GBA* and *POLG*. *GBA* L444P is a known risk factor for PD; in a study, it was shown to increase PD risk by 10 times (Sidransky et al., 2009). *POLG* mutations are linked to a wide range of systemic or neurological diseases (Stumpf et al., 2013). Although this gene mutation could rarely lead to parkinsonism, R964C has never been reported in association with parkinsonism thus far.

**Figure 4:**
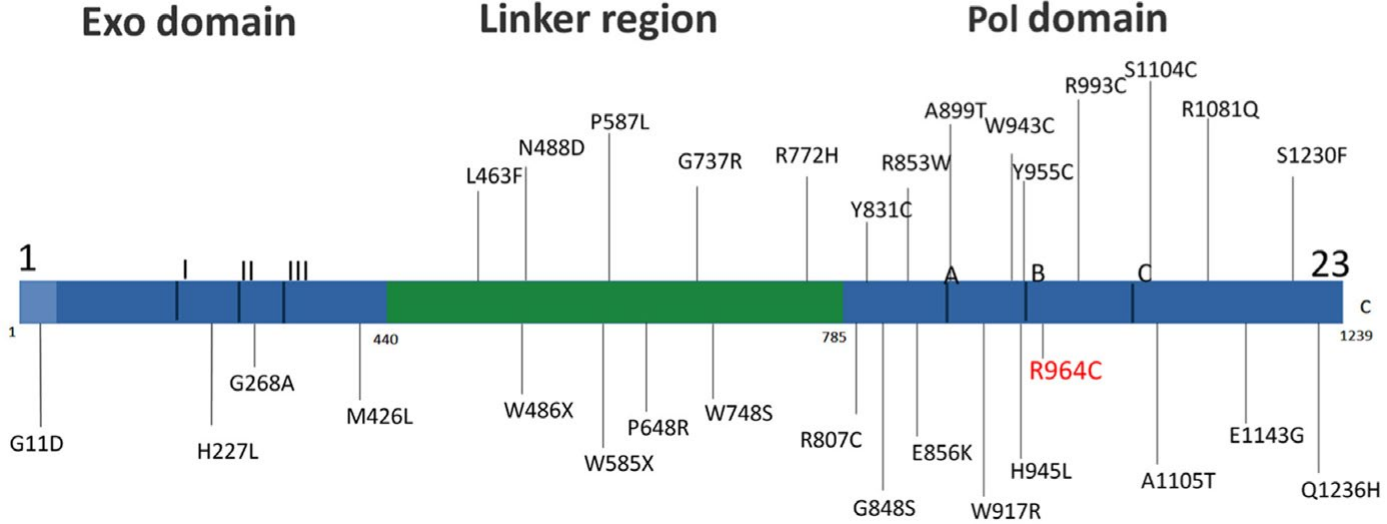
Mutation site in *POLG1* that potentially causes Parkinsonism

*POLG* mutation damages mitochondrial DNA (mtDNA), which lead to complex I respiratory chain dysfunction and depletion of mtDNA (Reeve et al., 2008; Stricker et al., 2009). *POLG* contains three domains: exonuclease (*exo*), linker region, and polymerase (*pol*; Figure 4) (Luoma et al., 2007). Table 1 summarizes the clinical features of patients carrying *POLG* mutations who had parkinsonism without progressive external ophthalmoplegia (Davidzon et al., 2006; Luoma et al., 2007; Mehta et al., 2016; Ylönen et al., 2013). According to Luoma et al., the *POLG pol* domain mutation might specifically present as parkinsonism (Luoma et al., 2004); the authors reported that seven families exhibited the parkinsonism‐related mutations over the *pol* domain. Parkinsonism may present in case of a *pol* domain mutation, but there were few gene mutations in other regions (Davidzon et al., 2006; Delgado‐Alvarado et al., 2015; Luoma et al., 2004; Mehta et al., 2016; Miguel et al., 2014; Mukai et al., 2013; Wong et al., 2008; Ylönen et al., 2013). Most *POLG* mutations have been found to be compound heterozygous missense substitutions or homozygous mutations, some of which still engendered clinical symptoms under heterozygous mutations in the *pol* domain. According to Murgai et al., heterozygous mutations can exhibit subclinical or milder manifestation, probably because of epigenetic regulation (Murgai & Jog, 2018). The *POLG pol* domain mutation may lead to parkinsonism as it aggravates oxidative stress in the dopaminergic neurons (Schapira & Gegg, 2011). Several imaging studies have indicated that patients with *POLG* mutations may exhibit severe and progressive loss of the dopaminergic neurons of the substantia nigra (Delgado‐Alvarado et al., 2015; Luoma et al., 2004; Tzoulis et al., 2013).

**Table 1:** *POLG* mutation phenotype without progressive external ophthalmoplegia

Genotype	Onset age of parkinsonsim	Family historys	Gender	Resting tremor	Rigidity	Bradykinesia	Seizure	Neuropathy	Levodopa response	Reference
G737R R853W	26	+	F	‐	+	+	‐	+	Good	Davidzon et al. (2006)
G737R R853W	20	+	F	‐	+	+	‐	+	Good	Davidzon et al. (2006)
R722H	57	−	F	+	+	+	−	−	Good	Luoma et al. (2007)
Y831C Q1236H	70	−	F	+	+	+	−	−	Good	Luoma et al. (2007)
R722H Q1236H	66	−	F	+	+	+	−	−	Good	Luoma et al. (2007)
S1230F	65	−	M	+	+	+	−	−	Good	Luoma et al. (2007)
P587L W748S	49	−	M	−	+	+	−	−	Good	Ylönen et al. (2013)
Y831C R722H	56	−	F	+	+	+	−	−	Good	Ylönen et al. (2013)
W748S R993C E1143G	72	−	F	+	+	+	−	−	Good	Ylönen et al. (2013)
E856K	18	+	M	−	+	+	−	−	Good	Mehta et al. (2016)
E856K	19	+	F	−	+	+	−	+	Good	Mehta et al. (2016)
R964C, GBA L444P	39	+	M	+	+	+	−	−	Good	Our case
R964C, GBA L444P	70	+	M	+	+	+	−		Good	Our case

The R964C mutation is located in the *pol* domain (Figure 4). Four studies have mentioned R964C mutation so far (Table 2). Homozygous R964C mutation can present as early ovarian failure or nucleotide reverse transcriptase inhibitor toxicity when anti‐human immunodeficiency virus‐1 medication is taken (Bailey, Kasiviswanathan, Copeland, & Anderson, 2009; Chen et al., 2018; Yamanaka et al., 2007). In two other studies, both compound heterozygous mutations at R964C and A862T were identified and revealed to be associated with ataxia, epilepsy, and intellectual disability (Table 2) (Stricker et al., 2009; Wong et al., 2008). According to its biochemical effect, R964C missense mutation can significantly reduce the catalytic efficiency compared with its wild type (Bailey et al., 2009). The recombinant R964C Pol γ activity had only 14% polymerase activity compared to Wide type. In the presence of nucleoside reverse transcriptase inhibitor, both heterozygously and homozygously harboring mutant R964C Pol γ lymphoblastoid cell lines contained significantly reduced mtDNA levels, compared with those wild type Pol γ (Yamanaka et al., 2007).

**Table 2:** *POLG* R964C mutation phenotype

Phenotype	Clinical manifestation	Onset age	Lactate acidosis	Epilepsy	Ataxia	Sensory neuropathy	PEO	Reference
Homozygous R964C mutation	NRTI toxicity		+++					Bailey et al. (2009), Yamanaka et al. (2007)
Homozygous R964C mutation	Nonsyndromic Ovarian dysfunction	34		−	−	−	−	Chen et al. (2018)
Compound heterozygous R964C and A862T mutations	ANS	6 and 15	+	+++	+	+	−	Stricker et al. (2009)
Compound heterozygous R964C and A862T mutations	ANS	17	−	+	+	+	−	Wong et al. (2008)
Heterozygous R964C and GBA L444P mutations	Parkinson's disease	39	−	−	−	−	−	Our case
Heterozygous R964C and GBA L444P mutations	Parkinson's disease	70		−	−		−	Our case

Note

PEO: Progressive external ophthalmoplegia, ANS: Ataxia Neuropathy Spectrum, NRTI: nucleotide reverse transcriptase inhibitor.

On the other hand, *GBA* mutation is known as loss of lysosomal hydrolase glucocerebrosidase (GCase) activity causing impairment of the autophagy lysosome pathway. Dysfunction of the mitophagy can be caused by impairment of autophagy lysosome pathway (Gegg & Schapira, 2016; Kim, Rodriguez‐Enriquez, & Lemasters, 2007). In animal model, heterozygous *GBA* L444P mutation mice exhibited reduction in GCase activity and impairment autophagic delivery of mitochondria to lysosomes and mitochondrial priming dysfunction (de la Mata et al., 2015; Li et al., 2019).

Moreover, accumulating evidence has indicated that harboring more than two mutational loci in two alleles may cause a synergetic effect, leading to early neurodegeneration (Cady et al., 2015; Giri, Zhang, & Lü, 2016). Also, polygenic factors contribute to the impairment of mitochondrial replication and repair may result in PD (Gaare et al., 2018). We suspect that these two gene mutations could both influence repairing mitochondria and increase oxidative stress causing early neurodegeneration.

In our patient's family, only one patient developed YOPD, whereas his father developed late‐onset PD. No literature has reported PD in *POLG* R964C mutation. Furthermore, the same mutations could reveal variable presentations, suggesting that epigenetic or environmental factors, as well as other modifiers may influence the clinical manifestation.

## 4 CONCLUSION

We reported a first familial PD of combined *POLG* R964C and *GBA* L444P mutations. This finding extends our understanding of the PD genotype–phenotype correlation.

## CONFLICT OF INTEREST

The authors declare no conflict of interest.
